# Regulation of Metastasis in Ewing Sarcoma

**DOI:** 10.3390/cancers14194902

**Published:** 2022-10-07

**Authors:** Mingli Li, Chunwei Chen

**Affiliations:** 1 Department of Systems Biology, Beckman Research Institute, City of Hope, Duarte, CA 91010, USA; 2 City of Hope Comprehensive Cancer Center, Duarte, CA 91010, USA

**Keywords:** Ewing sarcoma, fusion protein, EWSR1-FLI1, EWS-FLI1, heterogeneity, metastasis, prognostic markers

## Abstract

**Simple Summary:**

Ewing sarcoma (EwS) is the second most common bone and soft tissue cancer which mainly happens in children and adolescents. Currently, EwS patients are treated with a combination of surgery, radiation, and interval compressed chemotherapy. While outcomes have improved over the last several decades for patients with localized diseases, little progress has been made in the treatment of patients with newly diagnosed metastatic or relapsed diseases. Moreover, in localized cases, the survival rates are around 70% after five years and 30% after ten years. However, one third of patients develop metastatic tumor and, when metastasis is diagnosed, the five-year survival rates drop sharply to 25%. There is, therefore, urgent need to dissect the regulatory mechanism of EwS tumor metastasis and thus develop treatment strategies to treat metastatic diseases. Here, we reviewed the regulation of metastasis in EwS and hoped this can guide future studies on metastasis.

**Abstract:**

Ewing sarcoma (EwS) is a type of bone and soft tissue tumor in children and adolescents. Over 85% of cases are caused by the expression of fusion protein EWSR1-FLI1 generated by chromosome translocation. Acting as a potent chimeric oncoprotein, EWSR1-FLI1 binds to chromatin, changes the epigenetic states, and thus alters the expression of a large set of genes. Several studies have revealed that the expression level of EWSR1-FLI1 is variable and dynamic within and across different EwS cell lines and primary tumors, leading to tumoral heterogeneity. Cells with high EWSR1-FLI1 expression (EWSR1-FLI1-high) proliferate in an exponential manner, whereas cells with low EWSR1-FLI1 expression (EWSR1-FLI1-low) tend to have a strong propensity to migrate, invade, and metastasize. Metastasis is the leading cause of cancer-related deaths. The continuous evolution of EwS research has revealed some of the molecular underpinnings of this dissemination process. In this review, we discuss the molecular signatures that contribute to metastasis.

## 1. Introduction

Ewing sarcoma (EwS) is the second most common primary malignant bone tumor in children and young adults [1,2,3]. Currently, EwS patients are treated with a combination of surgery, radiation, and interval compressed chemotherapy. While outcomes have improved over the last several decades for patients with localized diseases, little progress has been made in the treatment of patients with newly diagnosed metastatic or relapsed diseases [3,4]. The five-year survival rate for patients with localized disease is 70–80%. However, due to its highly aggressive nature, up to one-third of patients suffer from metastatic, recurrent, or refractory tumors and face poor outcomes with only 10–30% long-term survival [5].

Genetically, EwS tumors have very few mutations and they are mainly characterized by the expression of somatic chromosomal translocations which fuse the 5′ end of the Ewing Sarcoma Breakpoint region 1 (EWSR1) gene with the DNA-binding domain of E-twenty-six (ETS) family of transcription factors. Among these, 10% to 15% of cases have EWSR1 fused with transcription factor ERG, ETV1, E1AF, or FEV, whereas 85% of cases have EWSR1 fused with Friend leukemia integration 1 (FLI1) transcription factor which gives rise to the EWSR1-FLI1 fusion protein [6,7,8,9]. Therefore, most of studies in the EwS research field have been focused on EwS induced by EWSR1-FLI1. Acting as an aberrant transcription factor, EWSR1-FLI1 binds to chromatins (either at the loci with GGAA microsatellites or the canonical ETS binding sites) [10,11,12,13,14,15,16]. At the epigenetic level, several studies have shown that EWSR1-FLI1 is able to change chromatin states by recruiting chromatin modifiers to chromatin, and thus modify gene expression [16,17,18,19,20,21,22]. At the transcriptional level, it is well-known that, due to its role in modifying epigenetic states, EWSR1-FLI1 alters gene expression, and it not only activates but also represses gene expression [23].

The origins of tumor heterogeneity are multifactorial, and contributing factors include genetic variation, stochastic processes, different microenvironments, and cell plasticity [24]. Apart from the EWSR1-FLI1 fusion, EwS has very few recurrent genetic mutations which are at low frequencies: TP53 (5–10%), CDKN2A (10–22%), and STAG2 (15–20%) [6,25,26] and few recurring abnormalities induced by gains in chr 8 (50%), chr 2 (25%), chr 1q (25%), and chr 20 (10–20%) [1,26,27,28]. Therefore, different from other types of tumors, EwS is thought to be homogeneous. However, several studies reported that the expression level of EWSR1-FLI1 in EwS cells is highly variable and dynamic, leading to significant intra-tumoral heterogeneity [29,30,31,32]. Cells with high expression of EWSR1-FLI1 (EWSR1-FLI1-high) proliferate quickly, whereas cells with low expression of EWSR1-FLI1 (EWSR1-FLI1-low) tend to migrate and thus generate metastatic tumors [29,31]. Metastasis is the hallmark of cancer that is responsible for the greatest number of cancer-related deaths [33]. Understanding the underlying mechanism of metastasis would, therefore, help to distinguish between poor and good prognosis EwS patients at diagnosis in clinics and to develop more effective therapies.

Previously, we reviewed the epigenetic and transcriptional signaling in EwS [23]. Here, we discuss the heterogeneity features of EwS caused by the fluctuation of EWSR1-FLI1 expression and summarize the molecular mechanisms underlying EwS metastasis.

## 2. Regulation of EwS Heterogeneity and Metastasis

**Figure 1:**
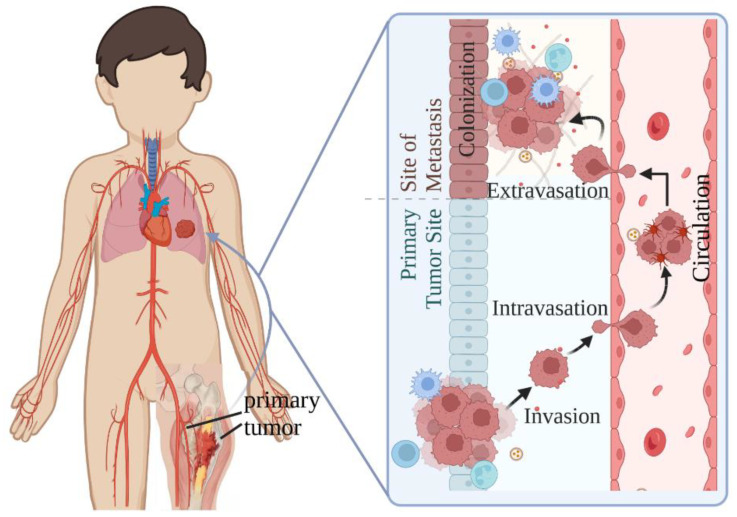
Metastasis of EwS tumors. EwS tumors metastasize to the lung, one of the most frequent metastasis sites of EwS tumors, through invasion, intravasation, circulation, extravasation, and colonization. The metastasis cascade figure was adapted from “overview of metastatic cascade”, by BioRender.com (accessed on 7 September 2022). Retrieved from http://app.biorender.com/biorender-templates (accessed on 7 September 2022).

**Figure 2:**
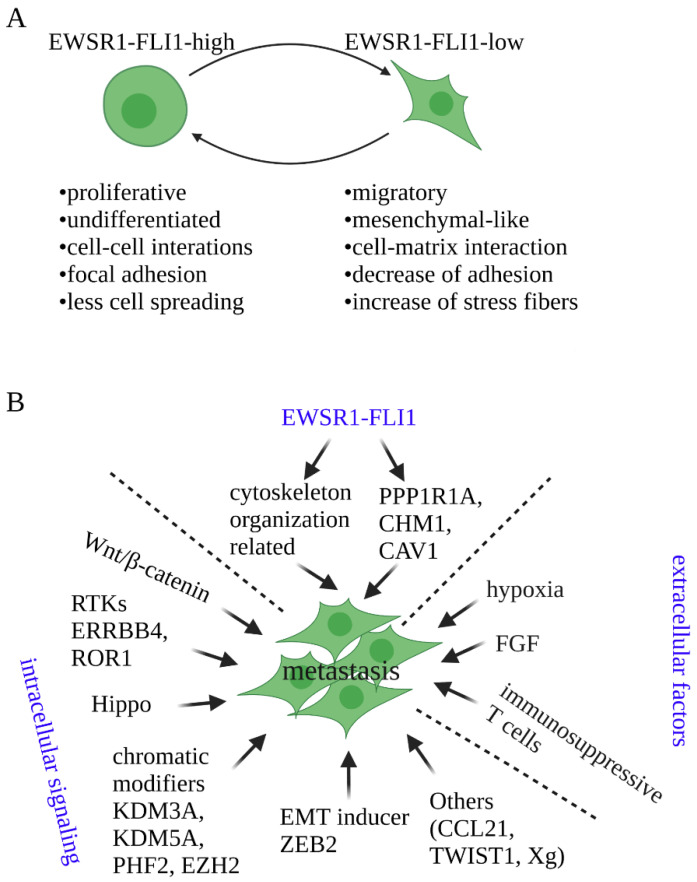
Signalings which contribute to EwS metastasis and features of EwS cells with different EWSR1-FLI1 expression levels. (**A**) Signaling that contributes to EwS metastasis. Both extracellular signalings offered by tumor micro-environment and intracellular signalings caused by gene expression changes are contributing to EwS metastasis. Extracellular signalings contributing to metastasis include FGF secreted from bone marrow stromal cells, hypoxia stress, and immunosuppressive T cells stored in the bone marrow. Intracellular signaling contributing to metastasis includes cellular signaling mediated Wnt, Hippo, receptor-tyrosine kinases (RTKs), chromatin modifiers KDM3A, KDM5A, PHF2, and EZH2, epithelia-to-mesenchymal transition transcription factor ZEB2, genes activated by EWSR1-FLI1 (PPP1R1A, CHM1, and CAV1), and genes encoding CCL21, TWIST1, and Xg. (**B**) Different statuses of EwS cells induced by distinct expression levels of EWSR1-FLI1. The expression level of EWSR1-FLI1 is different within and across distinct EwS cell lines and tumors. Cells with high expression of EWSR1-FLI1 are more proliferative and undifferentiated, whereas cells with low expression of EWSR1-FLI1 tend to have more mesenchymal-like features and have more migratory capacity.

Metastasis is a sophisticated multistep process that includes invasion that allows for the release of cells from the primary tumor site, intravasation that allows cells to enter the blood vascular, circulation of tumor cells along the blood cells, extravasation of tumor cells out of the blood vascular, and colonization of tumor cells to microenvironmental conditions encountered at the ectopic site (Figure 1) [34]. It is thus regulated by a large set of regulators and mechanisms. In EwS, it has been revealed that the expression of EWSR1-FLI1 is dynamic and the fluctuations in the EWSR1-FLI1 levels is critical for the metastatic capacity of EwS cells (Figure 2A) [29]. EWSR1-FLI1-high cells a have high proliferating propensity, whereas EWSR1-FLI1-low cells have a high metastatic tendency. Further, both extracellular signaling which is provided by surrounding tumor microenvironment and intracellular signaling which is offered by tumor cells themselves are contributing to the tumor metastatic development. The reported extracellular signaling includes hypoxia, growth factors, and immunosuppressive T cells in the bone marrow. The intracellular signaling can be grouped into several categories: chromatin modifiers [35], Wnt/β-catenin signaling [36,37,38], YAP/TAZ/TEAD axis [39,40], and receptor tyrosine kinases (RTKs) [41,42] (Figure 2B).

### 2.1. Roles of EWSR1-FLI1 in Modulating Heterogeneity and Metastasis

#### 2.1.1. Intra-Tumoral Heterogeneity Induced by the Fluctuation of EWSR1-FLI1 Expression

As introduced above, EwS is mainly driven by the expression of fusion protein EWSR1-FLI1 [5] and it has very few genetic mutations [6]. Therefore, it was considered to have a relatively homogenous context. However, several studies showed that EwS tumors also have significant heterogeneity [29,30,31,32]. Further, different from many other types of tumors whose heterogeneity is mainly caused by various mutations, intra-tumoral heterogeneity of EwS seems to be mainly induced by the dynamic fluctuation of fusion protein EWSR1-FLI1 expression [29,31]. Cells with high EWSR1-FLI1 expression (EWSR1-FLI1-high) proliferate in an exponential manner, whereas cells with low EWSR1-FLI1 expression (EWSR1-FLI1-low) have a strong propensity to migrate, invade, and metastasize (Figure 2B).

Variation of EWSR1-FLI1 expression within and across different EwS cell lines and primary tumors was firstly observed by a study conducted by Franzetti et al. [29]. Of note, these variable EWSR1-FLI1 expression levels correspond to the EWSR1-FLI1 activity which was indicated by the activity of EWSR1-FLI1 downstream targets. Of note, EWSR1-FLI1-low state and EWSR1-FLI1-high state are reversible, which explained the spontaneous presence of cells with different EWSR1-FLI1 expression levels in EwS tumors. Follow up study conducted by the same research group also explored the dynamics of individual cell transcriptomes induced by the variable EWSR1-FLI1 expression using single-cell RNA-seq [31]. In this study, a spectrum of transcriptional signatures was established using EwS cells with different EWSR1-FLI1 expression levels. Using this model system, the authors quantified the heterogeneity of EwS tumors and found that the variable EWSR1-FLI1 expression leads to EwS cells with different proliferation capacities, different oxidative phosphorylation levels, different hypoxia levels, and different glycolysis metabolism levels.

Recently, Khoogar et al. characterized the mechanism of EWSR1-FLI1 fluctuation using single-cell RNA-seq [32]. Upon EWSR1-FLI1 knockdown, the majority of EwS cells were directed into a quiescent-like state. However, a small portion of cells re-entered the proliferative cycles after prolonged EWSR1-FLI1 knockdown. Single-cell RNA-seq profiling analysis found that cells with EWSR1-FLI1 knockdown have different levels of EWSR1-FLI1 expression, and can be grouped into three populations, dormant population, transitional dormant population, and unperturbed population. The dormant population is the cells that entered the quiescent state, the transitional dormant population is the cells that re-entered the proliferative cycles after being quiescent for about 4–5 days, while the unperturbed population cells refer to the cells whose proliferation rates were not affected upon EWSR1-FLI1 knockdown. Gene expression analysis found that the cells in the transitional dormant population and unperturbed population express high levels of stem-like cell surface markers such as SOX2, CD40, and CELSR2. On the contrary, cells in the dormant population undergo autophagy. Furthermore, by comparing the expressing signatures between EwS cells and cells from different lineages, they found that EwS cells can be divided into three major cell types, embryonic stem cells (ESC), progenitor cells, and differentiated cells. In summary, the EWSR1-FLI1 expression level plays an important role in conferring cells with different cellular characteristics.

#### 2.1.2. EWSR1-FLI1 Controls Metastatic Potential by Regulating Cytoskeleton Organization and Altering Gene Expression

Cytoskeleton reorganization is an essential process for metastasis. Increasing evidence has revealed that modification of EWSR1-FLI1 expression level is sufficient to change the proliferation and migration properties of EwS cells by altering cytoskeleton organization [43,44,45]. For example, knockdown of EWSR1-FLI1 increased the expression of N-cadherin and therefore actin stress fibers [43]. Consequently, EwS cells change from small round cells with thin, short, and disorganized actin stress fibers to stretched cells with long and parallel actin stress, resulting in the acquisition of invasion capacity and an increase of cell migration [43]. Further transcriptomic and proteomic analysis found that EWSR1-FLI1 suppresses metastasis by repressing the expression of genes encoding focal adhesion, extracellular matrix, and actin regulatory proteins [45]. Of note, actin cytoskeleton and actin-based motility mediated by Rho is the main signaling pathway that is involved in EWSR1-FLI1-mediated cytoskeleton organization [29]. Moreover, EWSR1-FLI1 provides EwS cells proliferation property by up-regulating the expression of genes that are involved in cell-to-cell interaction but down-regulating the expression of genes that are associated with cell-matrix interaction [29].

In addition to the cytoskeleton organization-related genes, three other EWSR1-FLI1 directly downstream target genes have also been reported to be essential for metastatic colony generation. These three genes are protein phosphatase 1 regulatory subunit 1A (PPP1R1A) [46], BRICHOS chaperone domain-containing endochondral bone protein chondromodulin I (CHM1) [47], and caveolin-1 (CAV1) [48,49].

PPP1R1A was identified as a direct EWSR1-FLI1 downstream target by comparing the transcriptional profiles between EwS cells with and without EWSR1-FLI1 depletion [46]. Functions of PPP1R1A have been implied in glycogen metabolism, cardiac contractility, and cell proliferation [50]. In EwS, in addition to reduce the tumor progression, PPP1R1A knockdown reduced the metastases of xenografted EwS tumors, indicating that PPP1R1A can be used as a therapeutic target for metastasis treatment.

CHM1 was studied in EwS due to its function in mediating chondrogenic differentiation [47]. Compared to other types of cancer such as neuroblastoma and osteosarcoma, EwS has a higher expression of CHM1 [51,52]. The functional analysis found that the expression of CHM1 is transcriptionally induced by EWSR1-FLI1 [47]. Mechanistically, CHM1 mediates EwS progression especially metastasis and invasion by suppressing the differentiation potential of EwS. Downstream of CHM1, matrix metallopoptidase 9 (MMP9) acts as a mediator for CHM1-mediated metastasis.

Known as a protein that associates with cell surface caveolae, caveolin-1 (CAV1) plays roles in multiple cancer-associated processes e.g., cell migration and metastasis [53]. In EwS, CAV1 was initially identified as a direct target of EWSR1-FLI1, and it mediates the oncogenic phenotype and tumorigenicity of EwS tumors [48]. Further study found that CAV1 promotes EwS cells to migrate by activating MMP2 (matrix metalloproteinase-2) and up-regulating expression of MMP9, two zinc-containing proteolytic enzymes that cleave extracellular matrix protein [49,54]. Moreover, during the metastasis process, CAV1 helps EwS cells to colonize in the lung by transcriptionally inducing expression of SPARC (secreted protein acidic and rich in cysteine), a matricellular glycoprotein that mediates cell-to-cell interactions [54].

### 2.2. Extracellular Signaling Contributing to EwS Metastasis

#### 2.2.1. Hypoxia Stress-Induced Gene Expression Changes

As a tumor grows and expands, it eventually outreaches its blood supply, leading to a concurrent loss of nutrients and oxygen [55]. Hypoxia is, therefore, a fundamental micro-environmental component of solid tumor tissue. It has been revealed that hypoxia controls the malignant progression of solid tumors by activating hypoxia-inducible factor (HIF) transcription factors, HIF-1 and HIF-2, and thus altering gene expression [56,57,58,59].

In EwS, it has also been observed that, to respond to the micro-environmental stress, EwS cells transition to a more migratory and invasive cell state. For example, to respond to hypoxia stress, expression of HIF1α was identified in EwS [60,61,62]. Further study found that depletion of HIF1α led to an increase of cell proliferation, whereas hypoxia promotes anchorage-independent colony formation [61]. Phenotypically, hypoxia triggered matrix degradation and invadopodium formation in a Src-dependent manner [63]. At the molecular level, transcriptional profile analysis found that HIF-1α induces migration and metastasis by up-regulating genes associated with invasion and energy-producing metabolic pathways [62]. Further, hypoxia also increased the expression of EWSR1-FLI1 in a HIF-1α-dependent manner.

In addition to hypoxia, expression of HIF1α can also be induced by Y-box binding protein 1 (YB-1) [64,65,66], a spliceosome-associated factor which regulates RNA processing and translation [66,67]. Roles of YB-1 in promoting cell invasion, drug resistance, and malignant transformation have been observed in a wide variety of cancers [68,69]. Further, YB-1 induces epithelial-to-mesenchymal transition (EMT) by activating translation of mRNAs encoding EMT factors [70]. In EwS, compared to localized tumors, metastatic tumors have higher YB-1 expression [64]. Phenotypically, inactivation of YB-1 reduced motility of EwS cells in vitro and metastases to the lung in a in vivo xenograft mice model, highlighting roles of YB-1 in mediating metastasis. Mechanism characterization found that YB-1 promotes metastasis by activating translation of HIF1α under both normoxia and hypoxia. Targeting YB-1, class I HDAC inhibitor MS-275 inhibits YB-1 deacetylation, resulting in decreased binding of YB-1 on mRNA, and thus reduced protein translation and metastasis [65,66].

Downstream of hypoxia, Lawlor and colleagues found that hypoxia promotes metastasis of EwS cells through chemokine signaling [71]. CXCR4 (chemokine (C-X-C motif) receptor 4 (CXCR4)) is the most widely expressed chemokine receptor which induces chemotaxis upon binding to chemokines [72]. Together with its ligand CXCL12, CXCR4 plays critical role in cancer progression especially metastasis [73,74,75]. Similar to other types of cancer, in EwS, CXCL12 was found to be essential for neovascularization [76] and increased expression of CXCR4 was found to be associated with metastasis phenotype [77]. Further, EwS cells with high expression of CXCR4 have higher migration capacity, whereas inactivation of CXCR4 with pharmacologic inhibitor AMD3100 impaired the migration of EwS cells [71,78]. Consistently, in the clinic, CXCR4 expression correlated positively with the tumor sizes at diagnosis but correlated negatively with patients’ overall survival [78]. Downstream of CXCR4, it has been revealed that the small GTPases Cdc42 and Rac1 act as the key mediator of EwS metastasis.

#### 2.2.2. Growth Factors and Immunosuppressive T Cells in the Bone Marrow

Bone marrow is a large repository of many growth factors. Kamura et al. tested five different growth factors (FGF, IGF-1, PDGF, EGF, and TGF-b) and found that FGF increased EwS cell motility by activating FGFR1 signaling cascade [79]. This observation was further confirmed by the data showing the high levels of activated FGFR1 in the EwS biopsy samples [28]. Further tests found that FGF is secreted by bone marrow stromal cells (BMSCs) and FGF treatment led to morphological alterations of EwS cells. Downstream of FGFR1, PI3K is activated to change the cell morphology by activating Rac1, a small GTP-binding protein [79].

Another study conducted by Brinkrolf et al. investigated the pattern of immune cell subset distribution in the bone marrow of EwS patients [80]. They found that patients with localized EwS tumors have significantly lower numbers of immunosuppressive T cells than patients with metastasized EwS tumors. This observation indicated that local accumulation of immunosuppressive T cells in the bone marrow provides a supportive environment for EwS progression and metastasis, providing an immunotherapy strategy for EwS treatment.

### 2.3. Intracellular Signaling Contributing to EwS Metastasis

#### 2.3.1. Identification of the Gene Expression Signatures of Metastatic EwS at the Genome Level

To identify the specific gene expression signatures of metastatic EwS tumors, several studies compared the gene expression differences between localized tumors and metastatic tumors at the genome-wide level. For example, Ohali et al. analyzed the gene expression profiles of 14 primary tumor specimens and 6 metastatic tumor specimens from EwS patients using microarray [81]. From this study, they found that genes associated with metastasis and invasion are up-regulated in poor prognosis patients. Among these genes, cadherin-11 (OB-cadherin), a homophilic calcium-dependent cell adhesion molecule, and MTA1, a histone deacetylase, have been implied in metastasis of various cancer. Similarly, Schaefer et al. identified the signaling pathways that are involved in metastasis by profiling and annotating the differentially expressed genes in 7 metastatic tumors versus 20 localized tumors using microarrays [37]. From this study, 29 pathways including Wnt signaling, TP53, and NOTCH signaling were identified to be key metastasis-associated pathways in EwS.

Further, Zambelli et al. compared the molecular signatures of EwS cells with and without metastasis capacity at the genome level using microarray [82]. From this study, they found that high expression of LGALS3BP (lectin galactoside-binding soluble 3 binding protein) in EwS tumor cells is associated with a lower risk to develop metastasis and thus better event-free survival. LGALS3BP is a tumor-secreted antigen and a ligand of the lactose-specific S-type lectin, galactin-3 [83,84]. In contrast with EwS, in several other types of cancers including breast cancer [85], lymphoma [86], hepatocellular carcinoma [87], pleural mesothelioma [88], pancreatic cancer [89], and non-small cell lung cancer [90], up-regulated LGALS3BP expression in the tumor microenvironment but not tumor cells was observed and this upregulation is associated with poor survival outcomes. Further study in EwS found that LGAL3BP can be used as a prognostic marker to measure the metastatic propensity of EwS cells and patient outcomes.

Using two independent cohorts of clinically annotated EwS tumors, Volchenboum et al. compared the differentially expressed genes between EwS tumors derived from survivors and non-survivors and identified a list of genes to be associated with clinical outcomes [91]. Further, gene set enrichment analysis (GSEA) found that up-regulation of genes involved in the integrin pathway and chemokine receptor signaling is correlated with poor prognosis. Since integrin and chemokine signaling control the tumor-host interactions, this study emphasized the roles of the tumor environment in the modulation of tumor progression.

#### 2.3.2. Roles of Wnt/β-Catenin Signaling in Modulating EwS Metastatic Potential

Wnt/β-catenin signaling is best known for its function in mediating embryonic development and tissue homeostasis, and it is often deactivated in cancers [92,93]. Involvement of the Wnt signaling pathway in EwS metastasis was firstly recognized by a microarray analysis which compared the gene expression signatures between metastatic tumors and localized tumors [37]. As further evidence, high expression of LGR5, one of the receptors for Wnt ligands, was detected in a group of aggressive tumors [94] and DKK2, a Wnt signaling mediator, is associated with EwS tumor metastasis [51]. Furthermore, in the xenograft EwS tumor model, increased expression of β-catenin was observed in the invasive areas of metastatic tumors [95] and the patients with worse clinical outcomes are associated with activation of Wnt signaling. The functional analysis found that Wnt antagonizes EWSR1-FLI1 in altering gene expression, although the molecular mechanism of this antagonism has not been characterized [36]. Consequently, activation of Wnt promoted EwS cell migration and tumor metastasis in vivo.

Other mechanism studies have also found that Wnt regulates EwS metastasis by altering the expression of CXCR4, a well-known metastasis promoter in EwS [38,71,78] as described above. For example, Jin et al. observed that the expression of CXCR4 is regulated by Wnt5a [38], a non-canonical Wnt family representative, and a putative pro-metastatic factor [96]. Although expression levels of Wnt5a in different EwS cell lines vary, the expression level of CXCR4 positively correlates with the expression level of Wnt5a in all tested EwS cell lines. Further, exogenous expression of Wnt5a increased CXCR4 expression and thus EwS cell migration, whereas knockdown of endogenous Wnt5a reduced CXCR4 expression [38]. The mechanistic study found that induction of CXCR4 expression by Wnt5a is mediated by C-Jun N-terminal kinase (JNK) and protein kinase (PKC) [38]. These studies not only explained how Wnt signaling regulates metastasis in EwS, but also further delineated how CXCR4 signaling is regulated under the hypoxia stress condition as described above.

#### 2.3.3. Receptor Tyrosine Kinases (RTKs) in Modulating EwS Metastatic Potential

Receptor tyrosine kinases (RTKs) and their dysfunction have been applied in many oncogenic and tumor-promotion events [97,98]. The involvement of Erb-b2 receptor tyrosine kinase 4 (ERBB4) in EwS metastasis was identified by comparing the expression profiles of metastatic and non-metastatic EwS cell lines [41]. ERBB4 has higher expression in metastatic EwS cell lines, compared to non-metastatic EwS cell lines, and expression of ERBB4 is correlated with disease progression [41]. The roles of ERBB4 in mediating metastasis were further confirmed by data showing that knockdown of ERBB4 reduced metastasis in the xenograft model. Downstream of ERBB4, PI3K-Akt and focal adhesion kinase (FAK) [99], two genes associated with the migratory capacity of EwS cells, activate Rac1 GTPase to mediate ERBB4-promoted EwS cell invasion.

To systemically investigate the involvement of RTKs in EwS metastasis, Potratz et al. analyzed the expression of 55 of 58 RTKs [97,98] described in the human genome [42]. They found that 27 RTKs are over-expressed in EwS, especially metastatic EwS. In addition, compared to localized disease, 9 RTKs including receptor tyrosine kinase-like orphan receptor 1 (ROR1) have higher expression levels in metastatic disease, suggesting ROR1 contributes to metastasis. Indeed, the knockdown of ROR1 impaired the migration and invasion of EwS cells. Further, consistent with the studies showing the association of Wnt signaling in EwS metastasis [36,37,96], Wnt5a acts as the ligand for ROR1 to promote ROR1-mediated metastasis in EwS.

#### 2.3.4. Roles of Hippo/YAP/TAZ/TEAD Signaling in Modulating EwS Metastatic Potential

Kovar and colleagues characterized the roles of the Hippo/YAP/TAZ/TEAD axis-mediated signaling in modulating the metastatic propensity of EwS [39,40]. YAP (the Yes-associated protein 1) and its transcriptional co-activator TAZ (transcriptional co-activator with PDZ-binding motif) are the key downstream effectors of the Hippo signaling [39,100]. Upon activation by either Hippo signaling or Rho signaling, YAP/TAZ activates as a co-activator for TEA-domain transcription factor TEAD to initiate gene expression. In various cancers, over-expression and hyperactivation of YAP/TAZ have been associated with metastasis [101,102]. In a study characterizing the roles of EWSR1-FLI1 in reprogramming cytoskeleton and thus initiating metastasis, MRTFs (myocardin-related transcription factors), the Rho signal pathway transcriptional factor), were identified [39]. Compared to the putative EwS precursor mesenchymal stem cells, MRTFs-induced genes are suppressed in EwS primary tumors, suggesting EWSR1-FLI1 represses the expression of MRTFs. Moreover, knockdown of MRTFs in cells expressing shRNAs targeting EWSR1-FLI1 reversed the gene expression alterations induced by EWSR1-FLI1 knockdown, indicating that MRTFs are required for EWSR1-FLI1-mediated transcription. Interestingly, ChIP profiling revealed a significant overlap between MRTFs binding loci and EWSR1-FLI1 binding loci on the chromatin. Further, knockdown of EWSR1-FLI1 increased the numbers of MRTFs loci. These results suggest that EWSR1-FLI1 interferes binding of MRTFs on chromatin. Gene expression profiling found that MRTFs interact with TEAD at the TEAD motifs to regulate gene expression. Follow up studies showed that EWSR1-FLI1 also represses the expression of TAZ [40]. Consequently, the formation of YAP/TAZ/TEAD complex is disrupted by EWSR1-FLI1. Therefore, EWSR1-FLI1 utilizes a feed-forward inhibitory mechanism to inhibit the YAP/TAZ/TEAD axis to prevent metastasis [40]. Pharmacological inhibition of the YAP/TAZ/TEAD axis with verteoporfin (VP) blocked the EwS cell migration and invasion in vitro and EwS tumor metastasis in vivo [40]. YAP/TAZ/TEAD axis activity is low in non-malignant tissues, it therefore can be used to prevent metastasis without introducing severe side effects.

#### 2.3.5. Roles of Chromatin Modifiers in Modulating EwS Metastatic Potential

The involvement of epigenetic regulators in EwS metastasis was firstly reported by Jedlicka and colleagues who revealed that Jumonji-domain histone demethylase (JHDM) KDM3A regulates the metastasis of EwS tumor in the xenograft model [103]. From this study, MCAM (melanoma cell adhesion molecule) was identified as the key mediator for KDM3A-mediated metastasis.

Further, the Jedlicka research group studied the roles of JHDMs KDM5A and PHF2 in EwS metastasis [35]. Using the xenograft tumor model in vivo, they found that knockdown of KDM5A and PHF2 not only reduced the tumor burden in the primary tumor but also induced a more pronounced decrease in metastatic disease burden. The transcriptional analysis found that KDM5A and PHF2 promote metastasis by up-regulating the expression of genes that are normally repressed by EWSR1-FLI1. For example, KDM5A and PHF2 up-regulate MCAM and L1CAM, genes that are associated with the metastasis-promoting pathway in EwS [103], to promote metastasis. Considering the critical roles of KDM5A and PHF2 in the promotion of EwS metastasis, it is of interest to characterize the mechanism of KDM5A and PHF2 in antagonizing EWSR1-FLI1 function.

Another reported epigenetic regulator which regulates EwS metastasis is histone methyltransferase EZH2 [104]. Knockdown of EZH2 blocked the metastasized colony formation in the xenograft EwS model. Follow-up mechanistic study found that depletion of EZH2 induced differentiation of EwS cells, suggesting that EZH2 helps the metastasis by repressing the differentiation program of EwS.

#### 2.3.6. Transcription Factor ZEB2 Modulates EwS Metastasis

Zinc finger E-box binding homeobox 2 (ZEB2) is an epithelial-mesenchymal transition-inducing transcription factor that is consistently expressed in EwS patients [105]. Patients with metastatic disease have higher expression of ZEB2 than patients with no metastasis. ZEB2 knockdown displayed reduced migratory ability. The involvement of ZEB2 in EwS metastasis was further confirmed by a study that showed the association of protein phosphatase 1 regulatory subunit 1A (PPP1R1A) with metastasis [46]. As described above, PPP1R1A mediates EwS metastasis by acting as a direct downstream target of EWSR1-FLI1. Gene expression profiling analysis and functional annotation found that PPP1R1A regulated-genes are significantly overlapped with genes regulated by ZEB2. It would be of interest to characterize why PPP1R1A and ZEB2 have a similar function in modulating gene expression.

#### 2.3.7. Other Genes Involved in EwS Metastasis

Other reported genes which can be used as prognostic markers also include TWIST1 [106], CCL21 (chemokine C-C motif ligand 21) [107], and Xg [108]. TWIST1 is a transcription factor that is involved in normal neural crest development, and it has a similar function with ZEB2, a gene that has been reported to be associated with EwS metastasis [105]. Xg is a member of the CD99 family, a diagnostic marker of EwS tumor [109], whereas CCL21 has been used to increase dendritic cell-provoked T cell response in the clinic [110,111]. In EwS, high expression of TWIST1 and Xg was found to be positively correlated with poor survival outcomes [106,108], whereas low expression of CCL21 was observed in patients with metastatic disease and thus the expression level of CCL21 is negatively correlated with the patient survival [107]. Knockdown of TWIST1 or Xg significantly reduced lung metastasis, whereas forced expression of TWIST1 or Xg induced migration and invasiveness of EwS cells [79,106,108].

## 3. Discussion

Tumor cell heterogeneity is a key factor that contributes to drug resistance and tumor progression [24,112]. In EwS, intra-tumoral heterogeneity mainly comes from the fluctuation of EWSR1-FLI1 expression [29]. EwS cells switch between functionally distinct cell states dependent on dynamic fluctuations of EWSR1-FLI1 expression. EWSR1-FLI1-high cells proliferate in an exponential manner, whereas EWSR1-FLI1-low cells have a strong propensity to migrate, invade, and metastasize. Moreover importantly, EWSR1-FLI1-high and EWSR1-FLI1-low are reversible, enabling individual EwS cells to switch from proliferation to migration states. The heterogeneity and thus tumor cell plasticity may enable tumor cells to adapt to their environment by reversible adaptive phenotypic transformation and/or turning on and off specific markers. The fluctuation of EWSR1-FLI1 expression is, therefore, an intrinsic characteristic of its oncogenic potential. Characterization of the underlying mechanism of the fluctuation of EWSR1-FLI1 expression may provide more potential therapeutic strategies for EwS treatment.

Metastasis is one of the most critical adverse processes for successful cancer therapy [34], which is also true for EwS. Despite the remarkable paucity of somatic mutations, due to its high tendency to recur following resection and metastasize to other organs in which lungs are the most frequent metastasis sites, EwS is a very aggressive cancer [1,35]. EwS patients with metastatic disease have a poor survival rate. Disease management for patients with the localized disease has substantially improved but the prognosis for those with the metastatic or recurrent disease has changed very little over the past three decades. Five-year event-free survival for patients with metastatic disease is only 20% and curative therapy does not exist for patients whose disease recurs rapidly following therapy for localized disease. A better understanding of the cellular machinery and molecular mechanism contributing to tumor cell dissemination and metastasis is, therefore, essential to assess the potential prognostic value and to advance therapies to treat progressive disease in this high-risk population. For example,

## 4. Conclusions

The fluctuation of EWSR1-FLI1 expression plays an important role for the intra-tumoral heterogeneity. By switching between EWSR1-FLI1-high and EWSR1-FLI1-low states, EwS cells also use this mechanism to generate resistance for drug treatment and, more importantly, metastasize. Metastasis is a critical factor which is responsible for greatest number of cancer-related deaths. In EwS, in addition to gene expression changes induced by EWSR1-FLI1, multiple extracellular and intracellular factors have found to be critical for metastasis. However, due to the lack of in vivo model systems, study of EwS metastasis is still at the very initial stage. There is, therefore, critical need to dissect the mechanism of EWSR1-FLI1 fluctuation, look for alternative strategies for EwS treatment, and study regulation of metastasis in a more comprehensive setting.
